# Prevalence and risk factors of anxiety and depression among the community-dwelling elderly in Nay Pyi Taw Union Territory, Myanmar

**DOI:** 10.1038/s41598-021-88621-w

**Published:** 2021-05-07

**Authors:** Su Myat Cho, Yu Mon Saw, Thu Nandar Saw, Thet Mon Than, Moe Khaing, Aye Thazin Khine, Tetsuyoshi Kariya, Pa Pa Soe, San Oo, Nobuyuki Hamajima

**Affiliations:** 1 Department of Healthcare Administration, Nagoya University Graduate School of Medicine, 65 Tsurumai-cho, Showa-ku, Nagoya, 466-8550 Japan; 2 Nagoya University Asian Satellite Campuses Institute, Nagoya, Japan; 3 Department of Community and Global Health, The University of Tokyo, 5th Floor, Medical Bldg. No. 3, 7-3-1 Hongo, Bunkyo-ku, Tokyo, 113-0033 Japan; 4 Medical Care Division, Department of Medical Services, Ministry of Health and Sports, Office No. 4, Nay Pyi Taw, 15011 Nay Pyi Taw Union Territory Myanmar; 5 Planning Unit, Department of Public Health, Ministry of Health and Sports, Office No. 47, Nay Pyi Taw, 15011 Nay Pyi Taw Union Territory Myanmar; 6 Department of Preventative and Social Medicine, University of Medicine, 30th Street, Between 73rd & 74th Streets, Mandalay, Myanmar; 7 Department of Neurology, Yangon General Hospital, Bo Gyoke Road, Lanmadaw Township 11131, Yangon, Myanmar

**Keywords:** Health care, Genetics research

## Abstract

Providing elderly mental healthcare in Myanmar is challenging due to the growing elderly population and limited health resources. To understand common mental health problems among Myanmar elderly, this study explored the prevalence and risk factors of anxiety and depression among the elderly in the Nay Pyi Taw Union Territory, Myanmar. A cross-sectional study was conducted among 655 elderly by face-to-face interviews with a pretested questionnaire. Descriptive analysis and multiple logistic regression analyses were performed. The prevalence of anxiety and depression were 39.4% (33.5% for males and 42.4% for females) and 35.6% (33.0% for males and 36.9% for females), respectively. The adjusted odds ratio of having anxiety was significant for having low education level, having comorbidity, having BMI < 21.3, poor dental health, no social participation, and having no one to consult regarding personal problems, while that of having depression was significant for having comorbidity, having BMI < 21.3, poor vision, and having no one to consult regarding personal problems. The reported prevalence of anxiety and depression indicate the demand for mental healthcare services among Myanmar elderly. Myanmar needs to improve its elderly care, mental healthcare, and social security system to reflect the actual needs of its increasing elderly population. Screening for anxiety and depression among elderly with comorbidities should be promoted. Raising community awareness of mental health, encouraging social participation, and supportive counselling are also essential in combating anxiety and depression among Myanmar elderly.

## Introduction

Increasing elderly population is a global phenomenon, and the proportion of the global population over 60 years is estimated to double from 12.0% in 2015 to 22.0% in 2050^1^. In 2017, the number of elderly aged 60 years and above was 962 million worldwide and it is expected to be 2 billion by 2050^2^. In a similar fashion, the elderly population in Asia is expected to increase from 549.2 million in 2017 to 1.3 billion by 2050^2^.

Together with the rising elderly population, mental healthcare among the elderly has become a challenge for every nation as it can severely burden the health system, social security system, and economy both globally and nationally, as well as burden an individual's well-being^1,3,4^. One out of five individuals aged 60 years and above reported having mental or neurological disorders, and these disorders accounted for 6.6% of all disability-adjusted life years (DALYs) among individuals over 60 years^1^. Nearly 7.0% of the global elderly population had depression^1^, the third leading Level 3 cause of years lived with disability (YLD) counts for all sexes according to the 2017 Global Burden of Disease Study^5^. Approximately 4.0% of the elderly population worldwide suffered from anxiety disorders^1^, contributing 14.6% of DALYs caused by mental and substance use disorders^4^, Moreover, depression and anxiety among the elderly were unnoticed by the individuals themselves and were also under-diagnosed by healthcare professionals due to the misconception that these are normal parts of aging^1,5^. It could negatively aggravate several aspects at the individual, household, national, and international level^3–8^.

Myanmar, a lower-middle income country in South-East Asia, is struggling for healthy ageing with its growing elderly population and its limited resources for health and social security^9^. According to the latest census in 2014, Myanmar's total population expanded at an annual rate of 1.4%, while the elderly aged 60 years and above increased annually at 2.4%, exceeding the total population growth rate^10^. In 2014, the elderly aged 60 years and above comprised 8.9% of the national population^10^. The life expectancy at birth were 72.2 years for females and 64.9 years for males, while the healthy life expectancy at birth were 62.4 years for females and 57.4 years for males^4^. The index of ageing was 31.1 and the older age dependency ratio was 14.2 in 2014, which were higher than those of Indonesia, Cambodia, Laos, Philippines, and Timor-Leste^10^. It was projected that the elderly aged 60 years and above would account for 10.3% of the total population in 2020, and it was estimated to be doubled (20.2%) by 2050^10^. As Myanmar’s population pyramid has been shifting from expansive type^10,11^, Myanmar needs to improve its elderly healthcare, mental healthcare, health insurance system and social security system timely for its expanding elderly population.

Among Myanmar's long list of prioritised service needs, elderly mental health is given relatively less attention due to limited resources. Despite recent improvements in Myanmar’s healthcare, mental health expenditure, workforce, and service coverage are still challenges faced across the nation^9,12^. In 2017, the mental health expenditure was 0.36% of the total government health expenditure^13^, and there were only 627 mental health professionals^12^ working in the public sector—the main health service provider in Myanmar^9^. The numbers of psychiatrists and mental health workers per 100,000 people were 0.38 and 1.2, repectively^12^. Regarding inpatient mental healthcare, there were 22 psychiatric units in general hospitals, one forensic inpatient unit, and two mental hospitals in Myanmar^12^. For outpatient mental healthcare, there were 33 outpatient facilities in hospitals, three outpatient facilities in the communities, and two outpatient facilities for children and adolescents. Considering the scantiness of mental health professionals and facilities in Myanmar^9,12^, depression and anxiety among the elderly can be under-diagnosed and left untreated. Studies reporting elderly mental health problems are also limited in Myanmar. Due to specific characteristics of Myanmar society and frail healthcare and elderly support systems of the country, risks and protective factors of anxiety and depression among Myanmar elderly could be different from the previous research findings around the world. For a better understanding of elderly mental health problems, this study aimed to investigate the prevalence and risk factors of anxiety and depression among the elderly in the Nay Pyi Taw Union Territory, Myanmar.

## Materials and methods

### Study area, design, and participants

A cross-sectional study was conducted in the Nay Pyi Taw Union Territory, Myanmar, from December 2018 to January 2019. Nay Pyi Taw Union Territory, the capital territory since 2006, is located in the central region of Myanmar^11,13^, and has the 5th highest population density in the country. It has the longest life expectancy at birth (67.7 years for both sexes: 71.6 years for females and 63.7 years for males) among all states and regions of Myanmar, and its total fertility rate is 2.4 when the national figure is 2.5^11^. Similar to the national rural population distribution, nearly 68% of the total population of Nay Pyi Taw Union Territory reside in rural areas^11,13^. The elderly aged 60 years and above accounts for 7.2% of its total population^13^. Using simple random sampling, four out of eight townships in the Nay Pyi Taw Union Territory were selected. For data collection, 11 rural health centres (RHCs) were chosen based on their accessibility and availability. Detailed information on study design and sampling procedures have been described elsewhere^14^.

The elderly participants who could understand Myanmar language, aged 60 years and above, lived in the survey area for more than 6 months, and were physically and mentally fit enough to participate in the study took part in this study. Of the 811 participants, 655 elderly (221 males and 434 females) were considered for the final analysis after deleting missing and incomplete responses to the outcome variables.

### Data collection

Face-to-face interviews were carried out at RHCs using a pre-tested questionnaire in Myanmar language. Socio-demographic characteristics, health-related characteristics, Geriatric Anxiety Inventory^15^ (GAI) for anxiety assessment, and Geriatric Depression Scale^16^ (GDS) for depression assessment were incorporated into the questionnaire. The height and weight of the study participants were measured by the trained research assistants.

### Geriatric anxiety inventory

GAI is not a diagnostic tool but a screening tool with good psychometric properties for identifying anxiety symptoms, specifically in the elderly population. This self-reported scale for anxiety symptoms with simple dichotomous scoring (Yes = 1/No = 0) was appropriate for primary care and acute geriatric care settings^15^. In this study, the 20-item GAI was used to measure anxiety. The sensitivity and specificity of this tool were 73.0% and 80.0%, repectively^15^.

### Geriatric depression scale

GDS is a reliable, valid, and widely used screening tool for depression in the elderly population^16–19^. Among various versions of the GDS, the 15-item GDS^17^, was used in this study with a sensitivity of 87.0% and a specificity of 83.0%^19^. The maximum total score for this version was 15: each item was scored one point for a positive response (Yes = 1, No = 0) with the exception of items 1, 5, 7, 11, and 13 (No = 1, Yes = 0).

### Outcome variables

The outcome variables of this study were anxiety and depression in elderly participants, measured by the 20-item GAI and the 15-item GDS, respectively. Regarding anxiety, elderly participants who scored 9 and above in the GAI were considered to have clinically significant anxiety. For depression, GDS scores were classified as follows: 0–4 as no depression, 5–8 as mild depression, 9–11 as moderate depression, and 12–15 as severe depression^8,18,19^.

### Independent variables

The independent variables consisted of individual information, social characteristics, and health-related characteristics: age, gender, marital status, education level, employment status, social participation, having someone to consult regarding personal problems, number of friends/relatives met per month, body mass index (BMI), vision, dental health, and comorbidity. Marital status was classified into three groups: single, married, and other (widowed/divorced/separated). Education level was categorised into low (illiterate/primary school level) and high (middle school level and above). Using the mean BMI (21.3), the BMI of the study participants was divided into two groups: BMI < 21.3 and BMI ≥ 21.3.

### Statistical analysis

The proportion of anxiety and depression were portrayed descriptively. The 95% confidence interval (95% CI) for the proportion were calculated based on a binomial distribution. Categorical variables were examined by chi-square tests. Multiple logistic regression analyses were conducted to explore the factors associated with anxiety and depression. Unadjusted odd ratios (UORs), adjusted odd ratios (AORs), and 95% confidence intervals (95% CI) were calculated, setting P-value < 0.05 as significance. IBM Statistical Package for the Social Sciences (SPSS) version 25.0 was used for data analyses.

### Ethical considerations

This study was reviewed and approved by the Institutional Review Board of the University of Public Health, Yangon, Myanmar (UPH-IRB 2018/Research/48) and the Ethical Review Committee of Nagoya University Graduate School of Medicine (15085). After thoroughly explaining the study's objectives, procedures, and the questionnaire contents, written informed consent was collected from the study participants or their proxy before the interview. The study was conducted according to the relevant regulations and guidelines. For the illiterate participants and those with poor vision, the data collectors read out and explained the content of the informed consent form for them. The privacy and confidentiality of the participants were attentively considered in every stage of the study.

## Results

The characteristics of the participants according to gender are shown in Table 1. Half of the total participants (49.8%) were aged 60–69 years. The majority of the participants (80.0%) had comorbidities. Females had a lower percentage in high education (9.2%) and a higher percentage of being dependent (72.8%). One-third of total participants (33.9%) were never involved in social participation, such as social gatherings, club activities, social and religious events, while 14.4% reported having no one to consult regarding their personal problems.

**Table 1:** Characteristics of the study participants by gender (N = 655).

Characteristic	Male (n = 221)		Female (n = 434)		Total (N = 655)	
	n	%	n	%	N	%
**Age (Mean = 70.2)**						
60–69	101	45.7	225	51.8	326	49.8
70–79	84	38.0	154	35.5	238	36.3
80–89	31	14.0	50	11.5	81	12.4
≥ 90	5	2.3	5	1.2	10	1.5
**Marital status**						
Single	13	5.9	28	6.5	41	6.3
Married	163	73.8	178	41.0	341	52.1
Other	45	20.4	228	52.5	273	41.7
**Education**						
High	61	27.6	40	9.2	101	15.4
Low	160	72.4	394	90.8	554	84.6
**Employment status**						
Employed	98	44.3	104	24.0	202	30.8
Dependent	98	44.3	316	72.8	414	63.2
Retired	25	11.3	14	3.2	39	6.0
**Social participation**						
Yes	155	70.1	278	64.1	433	66.1
No	66	29.9	156	35.9	222	33.9
**Having someone to consult personal problems**						
Yes	185	83.7	376	86.6	561	85.6
No	36	16.3	58	13.4	94	14.4
**Number of friends/relatives met per month**						
0–6	73	33.1	120	27.6	193	29.5
7–13	77	34.8	167	38.5	244	37.3
≥ 14	71	32.1	147	33.9	218	33.3
**Body Mass Index (BMI)**						
Underweight (BMI < 18.5)	56	25.3	126	29.0	182	27.8
Normal (BMI 18.5–22.9))	97	43.9	151	34.8	248	37.9
Overweight (BMI 23.0–27.4))	51	23.1	110	25.3	161	24.6
Obese (BMI ≥ 27.5)	17	7.7	47	10.8	64	9.8
**Vision**						
Good	113	51.1	209	48.2	322	49.2
Poor	108	48.9	225	51.8	333	50.8
**Dental health**						
Good	122	55.2	215	49.5	337	51.5
Poor	99	44.8	219	50.5	318	48.5
**Co-morbidity**						
No co-morbidity	47	21.3	84	19.4	131	20.0
At least one co-morbidity	108	48.9	179	41.2	287	43.8
Two and more co-morbidity	66	29.9	171	39.4	237	36.2

Table 2 illustrates the proportions of the participants with anxiety and depression by gender. Of the 655 participants, 258 participants (39.4%; 95% CI 35.6–43.2%) reported having anxiety, while 233 participants (35.6%; 95% CI 31.9–39.3%) reported having depression. Females were more likely to have anxiety compared to males, but no gender difference was reported for having depression. In total, 21.5% (19.0% of males and 22.8% of females) had mild depression, while 10.5% (10.9% of males and 10.4% of females) and 3.5% (3.2% of males and 3.7% of females) reported moderate and severe depression, respectively.

**Table 2:** Prevalences of anxiety and depression among the study participants by gender (N = 655).

Mental health problems	Male (n = 221)			Female (n = 434)			Total (N = 655)			P-value^¢^
	n	%	95% CI	n	%	95% CI	N	%	95% CI	
**Anxiety**										0.027
No anxiety (GAI score 0–8)	147	66.5	(59.9–72.7)	250	57.6	(52.8–62.3)	397	60.6	(56.8–64.4)	
Anxiety (GAI score ≥ 9)	74	33.5	(27.3–40.1)	184	42.4	(0.377–0.472)	258	39.4	(35.6–43.2)	
**Depression**										0.690
Normal (GDS score 0–4)	148	67.0	(60.3–73.1)	274	63.1	(58.4–67.7)	422	64.4	(60.6–68.1)	
Mild (GDS score 5–8)	42	19.0	(14.1–24.8)	99	22.8	(19.0–27.0)	141	21.5	(18.4–24.9)	
Moderate (GDS score 9–11)	24	10.9	(7.1–15.7)	45	10.4	(7.7–13.6)	69	10.5	(8.3–13.1)	
Severe (GDS score 12–15)	7	3.2	(1.3–6.4)	16	3.7	(2.1–5.9)	23	3.5	(2.2–5.2)	

P-value^¢^: P-value of the chi-square test between males and females.

*GAI* Geriatric Anxiety Inventory, *GDS* Geriatric Depression Scale.

In the adjusted analysis, having low education level (AOR 1.99; 95% CI 1.09–3.62), no social participation (AOR 1.76; 95% CI 1.23–2.52), having no one to consult regarding personal problems (AOR 1.84; 95% CI 1.14–2.95), having BMI < 21.3 (AOR 1.64; 95% CI 1.15–2.36), poor dental health (AOR 1.63; 95% CI 1.14–2.31), having at least one comorbidity (AOR 2.46; 95% CI 1.48–4.08), and having two and more comorbidities (AOR 3.44; 95% CI 2.02–5.86) were significantly associated with anxiety (Table 3).

**Table 3:** Multiple logistic regression of anxiety and associated factors among the study participants (N = 655).

Characteristic	Anxiety			
	UOR	95% CI	AOR	95% CI
**Age (Mean = 70**.**2)**				
< 70 years	1		1	
≥ 70 years	0.91	(0.67–1.25)	0.71	(0.49–1.04)
**Gender**				
Male	1			1
Female	1.46	(1.04–2.05)*	1.36	(0.91–2.04)
**Marital status**				
Married	1		1	
Single	1.38	(0.72–2.66)	1.44	(0.71–2.94)
Other	1.05	(0.76–1.45)	0.81	(0.55–1.19)
**Education**				
High	1		1	
Low	2.50	(1.52–4.10)***	1.99	(1.09–3.62)*
**Employment status**				
Employed	1		1	
Dependent	0.97	(0.69–1.37)	0.94	(0.62–1.41)
Retired	0.58	(0.27–1.22)	1.01	(0.42–2.44)
**Social participation**				
Yes	1		1	
No	1.95	(1.40–2.71)***	1.76	(1.23–2.52)**
**Having someone to consult personal problems**				
Yes	1		1	
No	2.13	(1.37–3.32)**	1.84	(1.14–2.95)*
**Number of friends/relatives met per month**				
0–6	1		1	
7–13	0.99	(0.67–1.46)	0.91	(0.60–1.39)
≥ 14	1.15	(0.78–1.71)	1.02	(0.66–1.56)
**BMI (Mean = 21**.**3)**				
BMI ≥ 21.3	1		1	
BMI < 21.3	1.46	(1.06–2.00)*	1.64	(1.15–2.36)**
**Vision**				
Good	1		1	
Poor	1.50	(1.10–2.06)*	1.31	(0.92–1.86)
**Dental health**				
Good	1		1	
Poor	1.80	(1.31–2.47)***	1.63	(1.14–2.31)**
**Co-morbidity**				
No co-morbidity	1		1	
At least one co-morbidity	2.42	(1.51–3.89)***	2.46	(1.48–4.08)***
Two and more co-morbidity	3.15	(1.94–5.12)***	3.44	(2.02–5.86)***

*AOR* Adjusted for age, gender, marital status, education, employment status, social participation, having someone to consult personal problems, number of friends/relatives met per month, body mass index, vision, dental health, co-morbidity; *UOR* unadjusted odd ratios, *BMI* Body Mass Index.

*p < 0.05, **p < 0.01, ***p < 0.001.

Table 4 describes the output of the multiple logistic regression of depression and associated factors among the participants. Depression was positively associated with having no one to consult regarding personal problems (AOR 4.23; 95% CI 2.57–6.95), having BMI < 21.3 (AOR 1.73; 95% CI 1.19–2.52), poor vision (AOR 1.70; 95% CI 1.18–2.45), having at least one comorbidity (AOR 2.18; 95% CI 1.29–3.69), and having two and more comorbidities (AOR 3.01; 95% CI 1.73–5.23).

**Table 4:** Multiple logistic regression of depression and associated factors among the study participants (N = 655).

Characteristic	Depression (N = 233)			
	UOR	95% CI	AOR	95% CI
**Age (Mean = 70**.**2)**				
< 70 years	1		1	
≥ 70 years	1.08	(0.79–1.49)	0.80	(0.54–1.17)
**Gender**				
Male	1		1	
Female	1.18	(0.84–1.67)	1.06	(0.70–1.62)
**Marital status**				
Married	1		1	
Single	0.68	(0.31–1.41)	0.78	(0.36–1.71)
Other	1.13	(0.81–1.57)	0.92	(0.62–1.37)
**Education**				
High	1		1	
Low	2.53	(1.51–4.25)***	1.67	(0.89–3.12)
**Employment status**				
Employed	1		1	
Dependent	1.17	(0.82–1.66)	1.16	(0.76–1.77)
Retired	0.50	(0.22–1.14)	0.85	(0.33–2.23)
**Social participation**				
Yes	1		1	
No	1.70	(1.21–2.37)**	1.42	(0.98–2.05)
**Having someone to consult personal problems**				
Yes	1		1	
No	4.67	(2.93–7.45)***	4.23	(2.57–6.95)***
**Number of friends/relatives met per month**				
0–6	1		1	
7–13	0.90	(0.61–1.35)	0.87	(0.56–1.35)
≥ 14	1.33	(0.89–1.98)	1.21	(0.77–1.88)
**BMI (Mean = 21**.**3)**				
BMI ≥ 21.3	1		1	
BMI < 21.3	1.60	(1.16–2.21)**	1.73	(1.19–2.52)**
**Vision**				
Good	1		1	
Poor	1.84	(1.33–2.54)***	1.70	(1.18–2.45)**
**Dental health**				
Good	1		1	
Poor	1.66	(1.20–2.29)**	1.35	(0.94–1.95)
**Co-morbidity**				
No co-morbidity	1		1	
At least one co-morbidity	2.15	(1.33–3.49)**	2.18	(1.29–3.69)**
Two and more co-morbidity	2.64	(1.62–4.31)***	3.01	(1.73–5.23)***

*AOR* Adjusted for age, gender, marital status, education, employment status, social participation, having someone to consult personal problems, number of friends/relatives met per month, body mass index, vision, dental health, co-morbidity; *UOR* unadjusted odd ratios; *BMI* Body Mass Index.

*p < 0.05, **p < 0.01, ***p < 0.001.

## Discussion

There were a few studies^8,20^ reporting geriatric depression but none reported the prevalence and risk factors of geriatric anxiety in Myanmar. To our knowledge, this study is the first to report the prevalence of both geriatric anxiety and depression, together with their risk and protective factors in this area of Myanmar. The prevalence of anxiety and depression among Myanmar elderly were 39.4% (33.5% of males and 42.4% of females) and 35.6% (33.0% of males and 36.9% of females), respectively. Females were more likely to have anxiety compared to males. Regarding anxiety, having low education level, no social participation, having no one to consult regarding personal problems, BMI < 21.3, poor dental health, and comorbidity were positively associated in the adjusted analysis. Having no one to consult regarding personal problems, having BMI < 21.3, poor vision, and comorbidities were significantly associated with depression.

The prevalence of anxiety and depression reported in this study were higher than those among Malaysian elderly residing in rural areas^21^. A study from Myanmar, using the 15-item GDS scale, reported a relatively higher prevalence of depression (41.4%)^9^. This difference may be because it was conducted in homes for the aged in the urban area of Yangon, and the elderly living in institutes were more likely to have depression than the community-dwelling elderly^22^. Using the 4-item GDS scale, a study in 2016 reported that 16.0–56.0% of Myanmar elderly had depression depending on the threshold applied. Although no data on elderly suicide mortality in Myanmar have been reported, the suicide mortality rate per 100,000 population in 2017 was 7.8^12^. Being neglected for decades under the military regime, Myanmar’s health and social security systems have greatly suffered. The early detection and prompt treatment of common mental health problems such as anxiety and depression among the elderly become a problem. In turn, the under-diagnosed and untreated anxiety and depression among the elderly can markedly burden the country's health system^3^. Recently, Myanmar has launched new social protection schemes for the elderly^23^, however, the adequacy, equity, coverage, and sustainability of the social security system for the whole elderly population are questionable. Moreover, Myanmar does not have long-term care programs for the elderly yet. Although filial obligation and intergeneration support are still strong in Myanmar society^23^, the elderly of poor socio-economic status still suffer tremendously from the lack of long-term care, and weak social security and health systems. Additionally, homes for the aged in Myanmar usually refuse to accept those with mental problems. All these facts indicated that Myanmar needs strong commitment and effort to upgrade its health and social security system reflecting the actual needs of increasing Myanmar elderly. This study signified the demands of mental healthcare among the elderly in Myanmar, highlighting the early detection and interventions of anxiety and depression.

In this study, having lower education level (illiterate/primary school level) was a risk for having anxiety, and only 15.4% of the participants had attended middle school and above. Previous findings also reported a positive association of high education level with anxiety and depression^24^. A cross-sectional study from Poland, using the 30-item GDS, reported depression as a significant social and health problem among educationally active elderly^25^. Health knowledge and health perception influenced by the education level can affect healthcare-seeking behaviours among Myanmar elderly^24^. Hence, special attention should be given to the screening and diagnosis of anxiety and depression among Myanmar elderly with low education.

No association between employment status and anxiety or depression was reported in this study in contrast to others^26^. This may be because two-thirds of the participants identified themselves as “dependent” but many of them engaged in family businesses or local organisations as volunteers or part-time workers. These engagements could make them feel less anxious and depressed since they are able to contribute to their family and community^5,26^, and creating opportunities of social participation and social roles for the dependent elderly^5,26^. Another reason may be because the elderly are highly valued and are treated with respect regardless of their socioeconomic status in Myanmar society.

Having comorbidities was significantly associated with both anxiety and depression in this study. In total, 43.8% of the participants reported having at least one comorbidity, and 36.2% reported having two or more. Previous studies in Myanmar reported that unhealthy elderly individuals were more likely to be depressed^23^, and 57.9% of Myanmar elderly had at least one chronic condition, while 33.2% had two or more^27^. Non-communicable diseases (NCDs) are common among the elderly and two-thirds of Myanmar elderly reported having hypertension while 3.7% reported having diabetics^27^. To combat NCDs, Myanmar government has opened hypertension and diabetes clinics at RHCs every Wednesday in recent years. Considering that the majority of total population reside in rural areas, the public sector is the main health service provider, and with the country’s limited mental health specialists and infrastuctures^9,12^, these clinics could be an alternative channel for early detection of depression and anxiety if basic health staff could be trained for screening common mental health problems. Moreover, one of the misconceptions about the elderly is that anxiety and depression are normal parts of ageing. As depression and anxiety among the elderly are usually unnoticed by the elderly themselves and under-diagnosed by health professionals^1^, general practitioners and other health professionals in Myanmar should screen for depression and anxiety among elderly patients with multiple comorbidities. Myanmar elderly should also be informed of the overlapping nature of comorbidity and mental health problems to improve healthcare-seeking behaviours for both mental and physical illnesses.

This study reported that nearly two-thirds of the participants were either underweight, overweight, or obese, and having BMI < 21.3% was associated with anxiety and depression. Korean elderly presented more depressive symptoms in underweight BMI group compared to normal and obese ones^28^. Japanese researchers also revealed that being underweight decreased quality of life of the elderly^29^. Hence, maintaining proper body weight can be a protective factor for geriatric depression and anxiety.

In this study, half of the participants had poor vision and poor dental health. Elderly participants with poor vision were at risk for depression, while those with poor dental health were at risk for anxiety. Functional limitations such as vision impairment and dental problems among the elderly are regarded as part of the ageing process. Oral disorders ranked as the greatest age-standardized prevalence for both males and females in 2017^3^, influencing anxiety and depression among the elderly^30,31^. Many studies have pointed out the association of poor vision with depression and anxiety^5,6,24^. Vision problems were reported as a predictive factor for depression and anxiety and how it affects the quality of life of the elderly^5–7^. To reduce anxiety and depressive symptoms among the elderly, providing proper eye care and dental care for the elderly should not be neglected.

Although the number of friends or relatives met per month had no association with either anxiety or depression in this study, having someone to consult regarding personal problems was reported as a protective factor. Previous studies revealed the importance of supportive counselling, emotional support, and social participation in anxiety and depression among the elderly^5,6^. Elderly people may feel lonely and socially inactive in late life because of retirement, having fewer peers or friends as they get older, feeling less respected and powerless as they decline from their social roles, becoming financially or physically dependent on others, living alone or having less family time, and being stereotyped and stigmatised due to ageing. In Myanmar society, the elderly are highly respected and are mentors for the youth. It may be difficult for the elderly to find someone to consult their personal problems or to cry for helps and solutions when they have personal problems. Professional counselling services through phone calls, mobile applications, and the Internet are available nowadays, nevertheless, Myanmar elderly rarely use them^8^. Myanmar people prefer to cope with their mental crisis through religious practices (e.g. mindfulness meditation, confession at churches) than through professional helps^5,8^. Public awareness of mental health problems is still low, and stigmatisation of mental illness is still present in Myanmar.

This study reported that one-third of the participants were never involved in social participation, a protective factor for geriatric anxiety. Myanmar elderly usually participate in social and religious activities in their community, such as attending meditation sessions and Dharma talks, participating social or religious events at the temple, church, or mosque, and volunteering in local communities. In addition to their physical conditions and their dependency on others, stereotyping and stigmatisation of ageing by society or by themselves might also prevent Myanmar elderly from being socially active—as in other countries. Therefore, supports and encouraging for social participation by family members and their community are highly recommended.

In this study, two out of five females and one out of three males reported having anxiety. Marital status and age did not influence anxiety in this study. Depression was not found to be associated with age, gender, and marital status, similar to a previous Myanmar study^20^. Thai researchers reported that depression among the elderly was not associated with marital status, but females were more likely to have depression than males^24^. Poland researchers reported the associations of age and being female with depression but no association between marital status and depression^32^.

Regarding the study's limitations, the findings cannot be generalised for the whole nation, as it was conducted in only one out of fifteen administrative divisions. Recall bias may affect some responses. Only self-assessment tools were used to determine the presence of anxiety and depression, but diagnosis confirmation by psychiatrists was not performed. Further studies for in-depth understanding of the mental health of Myanmar elderly are highly recommended.

In conclusion, the prevalence of anxiety and depression among Myanmar elderly reported in this study indicate the demand for mental healthcare services for this population. Myanmar needs to improve its elderly care, mental healthcare, and social security system to reflect the actual needs of its increasing elderly population. Screening for anxiety and depression among Myanmar elderly with comorbidities should be promoted. Raising community awareness of elderly mental health, encouraging social participation, and supportive counselling are also essential in combating geriatric anxiety and depression in Myanmar.

## Data Availability

Data are available upon request from Department of Healthcare Administration, Nagoya University Graduate School of Medicine, Nagoya, Japan, for the researchers who meet the criteria for access. Researchers who would like to access to the data must contact Dr. Yu Mon Saw.
